# Challenges of Multidrug-Resistant Tuberculosis Meningitis: Current Treatments and the Role of Glutathione as an Adjunct Therapy

**DOI:** 10.3390/vaccines12121397

**Published:** 2024-12-12

**Authors:** Mohammad J. Nasiri, Kabir Lutfy, Vishwanath Venketaraman

**Affiliations:** 1 Department of Microbiology, School of Medicine, Shahid Beheshti University of Medical Sciences, Tehran 19839-69411, Iran; mj.nasiri@hotmail.com; 2 College of Pharmacy, Western University of Health Sciences, Pomona, CA 91766-1854, USA; klutfy@westernu.edu; 3 College of Osteopathic Medicine of the Pacific, Western University of Health Sciences, Pomona, CA 91766-1854, USA

**Keywords:** tuberculosis, mycobacterium tuberculosis, multidrug-resistant tuberculosis, tuberculosis meningitis, glutathione, antioxidants, adjunct therapy, immune modulation, cytokines, central nervous system, oxidative stress, immune response, therapeutic strategies, vaccines

## Abstract

Multidrug-resistant tuberculosis (MDR-TB) poses a significant global health threat, especially when it involves the central nervous system (CNS). Tuberculous meningitis (TBM), a severe manifestation of TB, is linked to high mortality rates and long-term neurological complications, further exacerbated by drug resistance and immune evasion mechanisms employed by Mycobacterium tuberculosis (Mtb). Although pulmonary TB remains the primary focus of research, MDR-TBM introduces unique challenges in diagnosis, treatment, and patient outcomes. The effectiveness of current treatments is frequently compromised by poor CNS penetration of anti-TB drugs and the necessity for prolonged therapy, which often involves considerable toxicity. This review explores the potential of cytokine-based adjunct immunotherapies for MDR-TBM, addressing the challenges of balancing pro-inflammatory and anti-inflammatory signals within the CNS. A central focus is the prospective role of glutathione, not only in reducing oxidative stress but also in enhancing host immune defenses against Mtb’s immune evasion strategies. Furthermore, the development of vaccines aimed at upregulating glutathione synthesis in macrophages represents a promising strategy to bolster the immune response and improve treatment outcomes. By integrating glutathione and innovative vaccine approaches into MDR-TBM management, this review proposes a comprehensive strategy that targets Mtb directly while supporting immune modulation, with the potential to enhance patient outcomes and reduce treatment related adverse effects. We underscore the urgent need for further research into adjunctive therapies and immunomodulatory strategies to more effectively combat MDR-TBM.

## 1. Introduction

Multidrug-resistant tuberculosis (MDR-TB) remains a major global health concern, particularly when it affects extrapulmonary sites such as the central nervous system (CNS) [1,2,3]. Tuberculous meningitis (TBM), a severe manifestation of TB, is associated with high mortality and long-term neurological complications, especially when associated with drug resistance [4,5]. TBM accounts for 5–10% of extrapulmonary TB cases and around 1% of all TB cases; however, these are likely underestimates due to limited microbiological confirmation and a lack of robust epidemiological studies [6]. While pulmonary TB continues to dominate research and treatment efforts, the emergence of MDR-TBM has introduced new complexities in diagnosis, treatment, and outcomes [7,8,9]. Studies indicate a significantly higher mortality rate in MDR-TBM compared to drug-susceptible TBM, with mortality rates ranging from 7.8% to 67%, depending on setting and drug resistance status, emphasizing the urgent need for more effective treatments for this severe form of TB [10,11,12].

In response to the MDR-TB crisis, the World Health Organization (WHO) released updated treatment guidelines, incorporating novel drugs bedaquiline (a diarylquinoline) and delamanid (a nitroimidazole), alongside shorter, more effective regimens for pulmonary MDR-TB [13]. These advancements have significantly improved the prognosis for pulmonary MDR-TB, offering hope for more effective management [14,15,16]. However, the management of TBM remains particularly challenging due to the limited penetration of many anti-TB drugs into the cerebrospinal fluid (CSF), leading to reduced efficacy in treating infections within the CNS [17]. MDR-TBM is further complicated by factors such as delayed diagnosis, the absence of optimized CNS-specific drug regimens, and prolonged treatment courses that often carry toxic side effects [18,19,20].

The unique pathogenesis of *Mycobacterium tuberculosis* (Mtb) within the CNS presents a significant challenge in managing MDR-TBM. Mtb in the CNS utilizes immune evasion mechanisms, such as resistance to oxidative stress, which allow it to survive within host cells and evade immune clearance. Recent research suggests that enhancing the host’s oxidative defenses through adjunctive glutathione therapy could provide a novel approach to countering Mtb’s survival strategies [21]. Glutathione, a critical antioxidant, not only bolsters immune responses but may also mitigate oxidative stress, potentially counteracting Mtb’s immune evasion mechanisms and improving patient outcomes.

This review examines current therapeutic approaches for MDR-TBM, evaluates the efficacy of newly recommended CNS-specific regimens, and explores promising adjunctive therapies such as GSH. By addressing both antimicrobial and immune modulation strategies, we aim to provide a comprehensive overview for improving MDR-TBM management and patient prognosis, aligning with recent insights into Mtb’s immune evasion tactics and the role of host oxidative defenses.

## 2. Current Treatment Options for MDR-TBM

MDR-TBM treatment is challenging due to the need for drugs that effectively cross the blood–brain barrier (BBB) while targeting resistant TB strains. Unlike pulmonary MDR-TB, which has standardized treatments, MDR-TBM lacks a consensus on optimal therapy [22]. This is due to the complex nature of TB meningitis and limited CNS penetration of many MDR-TB drugs, which reduces their efficacy against brain infections. Current approaches include modified regimens and research-driven strategies under evaluation for improved CNS effectiveness (Table 1).

### 2.1. Pretomanid-Based Combinations

Research has highlighted pretomanid’s promising penetration into the CNS, positioning it as a potential core drug in MDR-TBM regimens [23]. Studies demonstrated that pretomanid achieves higher concentrations in brain tissue compared to other MDR-TB drugs like bedaquiline and linezolid. Experimental regimens such as Pa_100SMx (pretomanid, sutezolid, moxifloxacin) have shown significant effectiveness in reducing bacterial load in the brain in animal models, which could make it an option for MDR-TBM in the future [23].

### 2.2. Modified WHO Regimens

The WHO’s guidelines recommend regimens for MDR-TB, and some studies have explored potential modifications to adapt these regimens for MDR-TBM. However, such adaptations remain experimental, and further research is necessary to confirm their efficacy and safety specifically for TB meningitis [23,24,25].

### 2.3. Ongoing Research

The WHO’s current guidelines and MDR-TB-focused trials, such as end TB and TB-PRACTECAL, emphasize shorter and less toxic treatments for pulmonary MDR-TB [22,26]. However, they do not directly address MDR-TBM, underscoring the need for further research and clinical trials to determine effective CNS-specific regimens for TB meningitis.

## 3. Challenges of Treatment Regimens in MDR-TBM

The evidence supporting MDR-TBM treatment is limited and largely extrapolated from pulmonary TB experience, presenting specific challenges when using MDR-TB regimens for TBM. A primary challenge is the limited drug penetration into the CNS.

Many drugs in MDR-TB regimens, such as bedaquiline and clofazimine, exhibit restricted CNS penetration, potentially reducing efficacy against MDR-TBM (Table 2) [24,27]. Bedaquiline reaches CSF, but at lower concentrations than in plasma, and data on its role in TBM treatment are scarce [28,29]. Similarly, pretomanid’s CNS penetration in the MDR-TB regimen is not well-documented, raising concerns about its impact on MDR-TBM [30,31]. Moxifloxacin achieves therapeutic levels in CSF at doses of 400 mg to 800 mg daily, but monitoring may be needed for MDR-TBM due to higher concentration requirements [32,33,34].

Linezolid shows better CNS penetration and is being evaluated with high-dose rifampicin, but it carries risks of severe side effects [36,37,38,39].

The prolonged treatment duration for MDR-TBM complicates standard regimens, as effective CNS clearance often requires extended therapy [42]. Key challenges include achieving adequate drug levels in the CSF, managing side effects, and adjusting treatment duration.

## 4. Adjunct Immunotherapies for MDR-TBM

**Figure 1:**
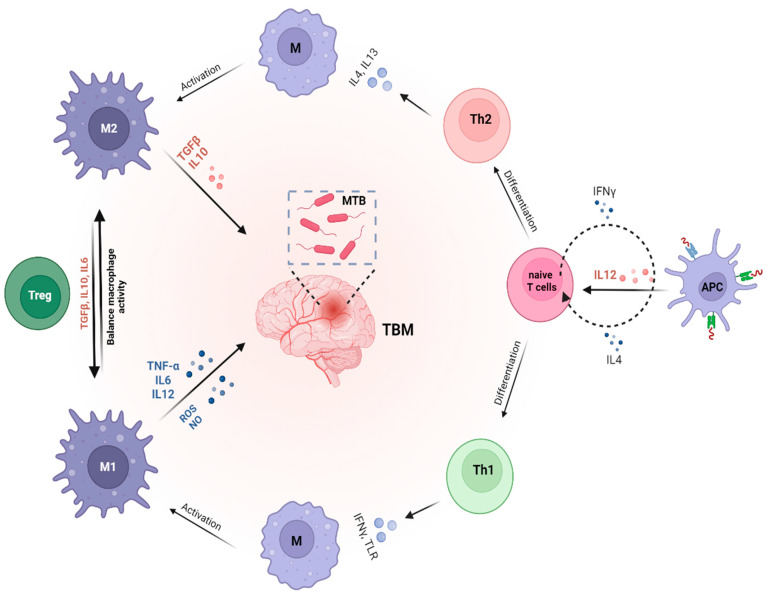
The role of cytokines in modulating immune response and Mtb susceptibility. Macrophages, which can differentiate into pro-inflammatory (M1) or anti-inflammatory (M2) subtypes, play a critical role in TBM by interacting with T cell subsets, including Th1, Th2, and regulatory T (Treg) cells. M1 macrophages are key for eliminating Mtb in the central nervous system, while M2 macrophages support tissue repair and regulate inflammation. Th1 cells, through cytokines such as IFN-γ, activate M1 macrophages to enhance the clearance of Mtb. In contrast, Th2 cells secrete cytokines like IL-4 and IL-13, promoting M2 macrophage polarization to facilitate tissue repair. Treg cells, by producing cytokines like IL-10 and TGF-β, help control excessive inflammation and maintain immune homeostasis.

The persistent challenges of MDR-TBM, such as poor treatment outcomes and limited CNS penetration of drugs, have spurred increased interest in adjunct immunotherapy options [43]. This strategy leverages the host immune system as a vital component for both containing and curing TB (Figure 1). However, while most studies focus on TB and MDR-TB, data specific to MDR-TBM remain limited, highlighting the need for further research in this area.

Using immunotherapy with cytokines alongside anti-TB treatment offers multiple potential benefits: it may improve treatment success rates, reduce treatment duration, and enhance the immune system’s ability to eliminate Mtb (Table 3). Cytokine immunotherapy can be divided into three categories based on their mechanisms of action: effector cytokine therapies, strategies for promoting a favorable immune response, and immunosuppressive strategies [43].

### 4.1. Effector Cytokine Therapies

These immunotherapies, which may be used for MDR-TBM, include recombinant IL-2, IL-7, IL-12, IL-15, IL-24, and IFN-γ, may enhance the immune response, improving microbicidal effects and overall management of TB and TBM.

Randomized controlled trials indicated that recombinant human IL-2, significantly improved sputum culture conversion and smear conversion in patients receiving at least three months of anti-TB therapy, including those with MDR-TB [44,45].

Similarly, trials on adjunctive therapy with IFN-γ, which could also be used for MDR-TBM, have demonstrated that both aerosolized and intramuscular administration can significantly improve sputum negative conversion rates and chest radiograph outcomes in patients with pulmonary MDR-TB [51,52].

In preclinical studies, recombinant adenoviruses IL-12 effectively reduced Mtb loads in infected mice [48], suggesting potential applicability for MDR-TBM. Additionally, in research on macaques infected with MDR-TB, IL-2 enhanced T-effector cell responses, leading to lower Mtb burdens and milder lung pathology [49].

Other studies found that mice supplemented with IL-7 and IL-15 exhibited a statistically significant reduction in mycobacterial burden in the lungs [47]. Moreover, both IL-7 and IL-15 were shown to enhance the survival of Mtb-infected mice [46], which could offer insights into new strategies for tackling MDR-TBM.

In an animal study using a mouse model infected with Mtb, exogenous IL-24 demonstrated protective effects by activating CD8+ T cells to produce IFN-γ through IL-24 receptors. Suppression of IL-24 may increase susceptibility to TB and contribute to chronic infection [50]. This highlights IL-24′s potential role in enhancing immune defenses, which may have implications for addressing MDR-TBM.

### 4.2. Promoting a Favorable Immune Response

Strategies such as using Toll-Like Receptor (TLR) agonists, like imiquimod, can effectively influence immune responses in TB. A recent study indicated that imiquimod significantly suppressed intracellular Mtb growth in macrophages through autophagy induction linked to increased nitric oxide production [53]. Moreover, imiquimod activate innate immune responses, stimulating macrophages and dendritic cells to release pro-inflammatory cytokines such as IL-12, which promotes Th1 differentiation and enhances Interferon-gamma (IFN-γ) production, further bolstering the body’s ability to combat Mtb.

A preclinical study also showed that anti-IL-4 neutralizing antibodies significantly reduced the Mtb burden in a murine model, indicating a shift toward a more effective Th1 immune response [54]. This suggests that targeting and suppressing the Th2 cytokine component in TB patients could enhance treatment outcomes. This suggests that targeting and suppressing Th2 cytokines in TB patients may shift the immune response toward a Th1 profile, enhancing IFN-γ production and macrophage activation. This shift could improve treatment outcomes, offering a promising strategy for managing MDR-TBM.

### 4.3. Immunosuppressive Strategies

Managing excessive inflammation in TBM is crucial. Corticosteroids, which lower Tumor Necrosis Factor-alpha (TNF-α) levels, are effective options for controlling inflammation in TBM. Multiple clinical studies indicated that corticosteroids, particularly dexamethasone, effectively reduce mortality in TBM. In a randomized, double-blind, placebo-controlled trial, patients receiving tapering doses of dexamethasone over eight weeks experienced a significant decrease in death risk [55]. Furthermore, long-term follow-up showed that adjunctive dexamethasone improved survival rates among TBM patients [56]. This suggests that adjunctive dexamethasone could similarly improve survival outcomes in MDR-TBM by addressing inflammation.

### 4.4. Balancing Pro- and Anti-Inflammatory Responses

Cytokine therapy for MDR-TBM must strike a delicate balance between enhancing the immune system’s ability to combat Mtb and controlling excessive inflammation that could damage the CNS. Clinical and preclinical studies provide valuable insights into how specific cytokines can be used to achieve this balance.

IFN-γ: In clinical trials for pulmonary MDR-TB, recombinant IFN-γ has been shown to activate macrophages, enhancing their ability to phagocytose and kill Mtb, thereby improving sputum conversion rates and reducing lung pathology. However, the strong pro-inflammatory response induced by IFN-γ necessitates careful management in CNS infections like MDR-TBM, where excessive inflammation can be harmful. In TBM, the co-administration of corticosteroids such as dexamethasone with IFN-γ can help manage this balance. Corticosteroids effectively lower TNF-α levels, reducing inflammation without compromising IFN-γ’s macrophage-activating properties. This combination has been shown to enhance clinical outcomes by promoting bacterial clearance while preventing inflammatory damage in TBM patients.

IL-10: As an anti-inflammatory cytokine, IL-10 has been explored in various clinical contexts, including autoimmune diseases such as Crohn’s disease and rheumatoid arthritis. IL-10 suppresses excessive immune activation by inhibiting the production of pro-inflammatory cytokines, offering a protective effect against tissue damage. In MDR-TBM, the controlled use of IL-10 could help mitigate the potentially damaging effects of pro-inflammatory cytokines like IFN-γ and TNF-α. Administering low doses of IL-10 in combination with Th1 cytokines may prevent neuroinflammation while preserving the immune system’s ability to control Mtb, providing a balanced therapeutic approach.

IL-12: Preclinical studies have demonstrated that IL-12 drives a robust Th1 immune response, reducing Mtb loads in animal models. However, clinical application of IL-12 must be carefully monitored due to its potent immune-activating effects, which can lead to excessive inflammation. In cancer immunotherapy, where IL-12 is used to stimulate anti-tumor immunity, it is often combined with immune checkpoint inhibitors to regulate immune activation. A similar approach could be applied in MDR-TBM, where IL-12 is paired with immunosuppressive agents to maintain an effective immune response against Mtb while avoiding overactivation that could damage CNS tissue.

TLR Agonists: TLR agonists such as imiquimod have been explored in clinical trials for infectious diseases and cancer. These agents stimulate innate immune responses by activating macrophages and dendritic cells, enhancing the production of pro-inflammatory cytokines like IL-12 and promoting a Th1 response. In MDR-TBM, TLR agonists could be used to trigger an initial immune response against Mtb. However, to prevent excessive inflammation in the CNS, TLR agonists could be combined with adjunctive anti-inflammatory treatments, ensuring that the immune response is potent enough to control the infection without causing harmful inflammation.

These clinical and preclinical examples highlight the importance of carefully regulating cytokine therapy to maintain a balance between pro-inflammatory and anti-inflammatory responses in MDR-TBM.

## 5. Challenges of Immunotherapies for MDR-TBM

While immunotherapies and cytokine therapies could aid MDR-TBM treatment, their application presents challenges that must be managed carefully. The complex immune environment, particularly in the CNS, requires precise control to prevent unintended effects. Below, we outline key obstacles and considerations necessary to advance cytokine use in MDR-TBM.

### 5.1. Balancing Challenges in Cytokine Therapy for MDR-TB

Cytokine therapy for MDR-TBM must be carefully balanced to manage the immune response effectively. The immune response against Mtb relies on a delicate equilibrium between pro-inflammatory and anti-inflammatory cytokines. Imbalances in this response could lead to adverse effects, making it essential that cytokine therapy is implemented cautiously to maintain this balance and prevent complications.

### 5.2. Limited Research in TBM-Specific Contexts

Most immunotherapeutic research has focused on pulmonary TB, leaving a significant gap in understanding TBM-specific responses. The interactions between anti-TB drugs and the immune system in MDR-TBM are not well understood, especially concerning how these drugs might affect the cytokine balance within the CNS. Anti-TB drugs may disrupt this balance, potentially exacerbating inflammation and worsening TBM outcomes. Further research is necessary to clarify how anti-TB therapies interact with immune responses in MDR-TBM [57,58,59].

### 5.3. Variability in Patient Response to Cytokine Therapy

There is considerable variability in individual responses to cytokine therapy due to factors such as genetics, pre-existing immune conditions (e.g., HIV, autoimmune diseases), and TB infection characteristics (e.g., drug susceptibility, drug resistance). Patients with drug-resistant TB often display different immune responses compared to those with drug-susceptible TB, underscoring the need for tailored immunotherapy approaches. To enable more targeted cytokine therapies, cytokine-release assays and immunomonitoring could be beneficial for assessing immune responses to Mtb antigens [60,61,62,63,64,65]. However, implementing these assays in TB management, particularly in MDR-TBM, is costly and challenging, as TB predominantly affects low-resource populations.

### 5.4. The Impact of Cytokines on the CNS Immune Environment

Using cytokines in MDR-TBM therapy presents unique challenges due to their pronounced effects on the CNS immune environment. Cytokine therapy in the CNS can influence bacterial metabolism and survival strategies. In MDR-TBM, these alterations may inadvertently support bacterial survival mechanisms, including the upregulation of efflux pumps and modifications to the cell wall structure. Both adaptations are associated with enhanced persistence of drug-resistant Mtb strains, complicating treatment and potentially promoting bacterial resilience [66,67,68].

## 6. Glutathione and MDR-TBM Management

**Figure 2:**
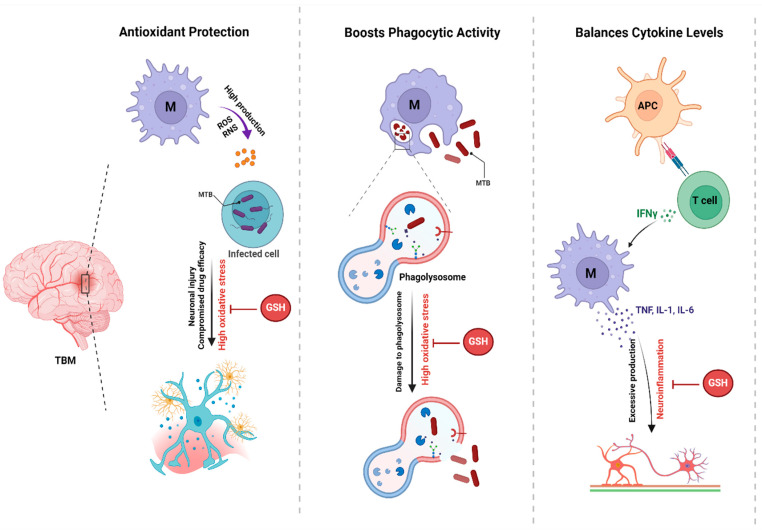
The Protective Role of Glutathione in TBM. This figure illustrates the mechanisms by which antioxidants contribute to host defense against Mtb infection. Antioxidants protect against oxidative stress, enhance phagocytic activity, and balance cytokine levels, all of which are crucial for effective immune responses.

As researchers continue to explore adjunct therapies for TBM, glutathione (GSH) has emerged as a potential candidate due to its critical role in modulating immune responses and protecting against oxidative stress in the CNS (Figure 2) (Table 4) [21].

GSH, which is composed of glutamine, cysteine, and glycine, is a powerful antioxidant that helps maintain cellular redox balance, particularly critical in TBM, where inflammation can lead to neuronal damage [21]. It supports lymphocyte and macrophage functions, enhancing immune responses against Mtb and potentially benefiting MDR-TBM management [69,70,71,72,73].

**Table 4:** Clinical Benefit of Glutathione in Managing MDR-TBM.

Benefit	Description	Mechanism	Clinical Relevance to MDR-TBM
Antioxidant Properties [21]	Glutathione acts as a key antioxidant, protecting cells from oxidative stress.	Maintains redox balance and reduces oxidative damage during inflammation, critical in TBM.	Protects neuronal integrity and function, which is vital in preventing neurological damage in TBM.
Immune Modulation [69,70,71,72,73]	Enhances immune response by improving lymphocyte and macrophage function.	Increases levels of Th1 cytokines (IFN-γ, TNF-α, IL-2) and reduces anti-inflammatory cytokines (IL-6, IL-10).	Strengthens host defenses specifically against MDR strains, improving treatment efficacy in MDR-TBM.
Synergistic Effect with NAC [71,74]	Combining glutathione with N-acetylcysteine (NAC) boosts intracellular levels.	NAC replenishes glutathione, enhancing its antioxidant effects and cellular protection.	May enhance the therapeutic response to standard TB drugs, addressing drug resistance in MDR-TBM.
Enhanced Bioavailability [75,76,77,78]	Liposomal formulations improve stability and delivery.	Encapsulates glutathione to protect against degradation, ensuring effective transport across the BBB.	Ensures effective delivery of antioxidants to the CNS, critical for managing TBM complications.
Prevention of Drug Resistance [74,79,80,81]	Cysteine (a glutathione derivative) prevents emergence of drug-resistant strains.	Inhibits cells from entering a persister state and induces an oxidative burst, enhancing drug activity.	Helps mitigate the development of drug resistance, a major concern in treating MDR-TBM.
Hepatoprotection [74,79,80,81]	NAC protects against hepatotoxicity associated with anti-TB drugs.	Reduces liver enzyme levels and mitigates drug-induced liver damage.	Essential for maintaining liver health, thus ensuring continuity of MDR-TBM treatment.
Cost-Effectiveness [82]	Could alleviate the financial burden on MDR-TB patients.	Enhances treatment efficacy and may reduce length of hospital stays.	Addresses the significant economic strain on patients, making treatment more accessible and sustainable.

MDR: Multidrug-Resistant, TB: Tuberculosis, CNS: Central Nervous System, NAC: N-acetylcysteine, BBB: Blood–Brain Barrier.

Advances in delivery systems, such as liposomal GSH, improve its bioavailability and CNS delivery, potentially enhancing its therapeutic impact on MDR-TBM cases where effective CNS penetration is critical [75,76,,77,78]. Beyond liposomal formulations, other advanced delivery strategies like nanoformulations, polymeric nanoparticles, and conjugated drug systems are being explored for their ability to enhance the CNS targeting of therapies. These systems can be designed to improve cellular uptake, stability, and the controlled release of glutathione, ensuring that it reaches therapeutic levels in the brain without causing systemic toxicity. Moreover, the use of cell-penetrating peptides or receptor-mediated transport systems may further enhance the selective targeting of liposomal GSH to CNS tissues, improving its efficacy in treating MDR-TBM. These advancements in delivery technologies are expected to significantly improve the clinical management of MDR-TBM, where high CNS concentrations of glutathione are necessary to combat inflammation, oxidative damage, and bacterial persistence in the brain.

The combination of GSH with NAC amplifies immune function by increasing Th1 cytokine production (IFN-γ, TNF-α) and decreasing IL-6 and IL-10 levels, which could be particularly beneficial in strengthening the immune response against MDR-TBM [71,74].

When used alongside standard anti-TB treatments, GSH and NAC enhance drug efficacy, reduce hepatotoxicity, and prevent drug resistance, positioning them as powerful adjuncts in MDR-TB and potentially in MDR-TBM management [74,79,80,81]. Additionally, GSH’s role in reducing treatment duration and hospital stays may alleviate financial burdens for MDR-TB patients, as 81% face catastrophic treatment costs [82]. These economic benefits could also extend to MDR-TBM patients, where prolonged and complex treatment further increases financial strain.

## 7. Glutathione and Oxidative Stress in MDR-TBM

To effectively manage MDR-TBM, understanding how GSH influences the host’s relationship with Mtb is essential. This involves examining oxidative stress mechanisms that allow Mtb to survive and evade immune responses, highlighting the importance of GSH in developing effective treatments for MDR-TBM.

One strategy Mtb employs to evade immune defenses is the synthesis of mycothiol, a GSH analog that protects the bacterium against oxidative stress [83,84,85]. This mycothiol-based defense is essential for Mtb’s persistence within infected cells. Mycothiol, a key thiol in Mtb, consists of glucosamine, myo-inositol, and cysteine, playing a vital role in detoxifying harmful substances and maintaining redox balance within the bacterial cell.

The synthesis of mycothiol begins with the enzyme mycothiol synthetase, which catalyzes its formation from N-acetylcysteine and glucosamine-1-phosphate [86,87,88]. Acting as an antioxidant like GSH in human cells, mycothiol neutralizes harmful compounds, enabling Mtb to evade immune defenses and survive within host cells, particularly in macrophages where oxidative stress poses a significant threat.

The interplay between host GSH and Mtb’s mycothiol is critical in TB therapy for MDR-TBM. Increased GSH levels in host cells can challenge Mtb’s defenses, as elevated GSH in macrophages may overwhelm mycothiol’s detoxification role, making Mtb more vulnerable to immune clearance. Additionally, higher levels of GSH can outcompete mycothiol in neutralizing harmful compounds, weakening Mtb’s ability to evade immune attacks and increasing its susceptibility to oxidative damage.

Glutathione also directly interferes with several key strategies Mtb employs to evade the host immune system. One major evasion tactic used by Mtb is the alteration of the host’s redox environment to resist oxidative damage. By increasing glutathione levels, the host can enhance the production of reactive oxygen species (ROS) and reactive nitrogen species, which create a hostile intracellular environment. Glutathione also influences macrophage function by promoting autophagy, a process where Mtb-containing phagosomes are engulfed and degraded. Elevated glutathione enhances the formation of autophagosomes, facilitating better bacterial clearance. Additionally, glutathione regulates key immune signaling pathways, including NF-kB and MAPK pathways, which control the release of pro-inflammatory cytokines like TNF-α and IL-12. These cytokines not only recruit immune cells to the site of infection but also activate macrophages and other immune cells, leading to an increased immune response. By modulating both oxidative stress and immune signaling, glutathione enhances the host’s ability to recognize and destroy Mtb, counteracting its immune evasion strategies. Moreover, glutathione’s role in maintaining redox homeostasis helps to prevent immune dysfunction, such as excessive inflammation, which could otherwise allow Mtb to survive and replicate within the host.

## 8. Implications of Glutathione for Vaccine Development

Insights into GSH’s potential to counteract Mtb immune evasion open new avenues in vaccine development for MDR-TBM. A targeted vaccine could aim to upregulate GSH synthesis within macrophages, where Mtb primarily resides, thereby creating an oxidative environment hostile to Mtb. This section explores specific strategies for developing such a vaccine (Table 5).

### 8.1. Upregulating Glutathione Synthesis via the Nrf2 Pathway

One promising approach is to stimulate the Nuclear factor erythroid 2–related factor 2 (Nrf2) pathway, a master regulator of antioxidant production, including GSH [89,90,91]. By including adjuvants or immunostimulatory molecules that activate Nrf2, a vaccine could promote the expression of genes responsible for GSH synthesis, such as glutamate-cysteine ligase, which catalyzes the rate-limiting step in GSH production. Enhancing Nrf2 activation would drive macrophages to produce higher GSH levels, increasing ROS and creating conditions that challenge Mtb’s ability to evade immune responses.

### 8.2. Increasing Availability of Glutathione Precursors

Another approach is to boost intracellular cysteine levels, a crucial precursor for glutathione production. A vaccine could include components that upregulate transporters like SLC7A11, enhancing cystine import into macrophages [92]. This steady supply of cysteine would sustain GSH synthesis within infected macrophages, supporting prolonged oxidative stress against Mtb and undermining its immune evasion mechanisms.

### 8.3. Enhancing NADPH Production for Sustained Glutathione Activity

To maintain GSH in its active, reduced form, an adequate NADPH supply is essential [93]. A vaccine could promote pathways that increase NADPH production by activating the pentose phosphate pathway or enhancing the expression of enzymes such as glucose-6-phosphate dehydrogenase. This steady NADPH supply would allow macrophages to maintain high levels of active GSH, strengthening their oxidative response and reducing Mtb’s capacity to survive under oxidative stress.

Moreover, these GSH-centric strategies could be integrated with other vaccine approaches targeting Mtb antigens or enhancing T-cell responses [94,95,96]. This multifaceted strategy could provide a more robust defense against MDR-TBM by not only enhancing the oxidative capacity of macrophages but also improving overall immune recognition and response to the pathogen.

## 9. Feasibility and Challenges of Glutathione-Targeted Vaccines for MDR-TBM

While the idea of leveraging glutathione synthesis in macrophages to combat MDR-TBM is promising, there are several practical considerations and challenges that must be addressed to translate this concept into viable vaccine strategies.

### 9.1. Practical Feasibility of Glutathione-Targeted Vaccines

Developing vaccines to enhance glutathione synthesis in macrophages requires identifying effective adjuvants or immunostimulatory molecules, such as Nrf2 activators. Key challenges include precise macrophage targeting to avoid systemic side effects and optimizing the bioavailability of adjuvants to activate Nrf2 without causing overactivation and excessive inflammation. Additionally, balancing glutathione precursor supplementation, particularly cysteine, is critical, as overstimulation could disrupt redox homeostasis, leading to potential immune cell damage. Careful dose optimization is essential to enhance oxidative stress against Mtb while minimizing immune pathology.

### 9.2. Potential Limitations and Side Effects

Upregulating macrophage glutathione raises concerns about chronic immune activation and its systemic effects. Excessive glutathione levels could lead to oxidative stress, potentially damaging host tissues, especially in individuals with compromised immune systems, such as those with autoimmune diseases or HIV. Additionally, tissue-specific variations in macrophage responses to glutathione modulation may affect vaccine efficacy. Targeting Nrf2 activation also carries risks, as excessive activation could disrupt immune balance, leading to autoimmune reactions or chronic inflammation. Therefore, controlling the timing and intensity of Nrf2 activation is crucial to minimize these risks.

### 9.3. Current Progress in Glutathione-Based Vaccine Research

While no vaccines targeting glutathione upregulation in macrophages have reached clinical trials, preclinical research is progressing. Nrf2 activators, being tested for diseases like cancer and neurodegenerative disorders, show promise in enhancing antioxidant defenses and may offer insights for TB immune modulation. In MDR-TBM, preclinical studies have shown that boosting glutathione synthesis enhances macrophage activity and pathogen clearance in TB mouse models. However, more research is needed to assess safety, efficacy, and side effects. Combining glutathione-based strategies with conventional TB vaccines may provide a more effective defense against MDR-TBM.

## 10. Future Research Directions

Future research on MDR-TBM should prioritize enhancing CNS drug delivery to improve the bioavailability of anti-TB medications, exploring innovative formulations like nanoparticles or liposomal carriers. Additionally, studies should focus on effectively boosting GSH levels in the CNS macrophages through Nrf2 pathway modulation or cysteine supplementation, while investigating the role of elevated GSH in immune responses. Cytokine-based immunotherapy also needs further exploration to identify optimal combinations that balance inflammatory responses and interact with oxidative stress modulators. Moreover, vaccine development should aim to enhance GSH synthesis and immune responses against Mtb’s redox balance, utilizing suitable adjuvants. Finally, clinical trials assessing new treatment combinations, drug formulations, and patient-specific factors are crucial for developing personalized approaches to improve outcomes in MDR-TBM. Understanding the variability in patient responses, along with optimizing GSH dose, administration, and its combination with other drugs and vaccines, will be key to tailoring therapeutic regimens effectively. Careful consideration of therapeutic regimens that incorporate GSH supplementation, its precise dosing, and how it synergizes with anti-TB drugs and vaccines will enhance treatment efficacy and minimize side effects. Tailoring these strategies based on individual patient profiles will be essential for achieving better treatment outcomes in this challenging disease.

## 11. Conclusions

MDR-TBM remains a challenging and life-threatening condition, worsened by limited CNS penetration of conventional anti-TB drugs and Mtb’s immune evasion mechanisms. This review discusses strategies to overcome these barriers, such as enhancing GSH synthesis and developing cytokine and vaccine-based immunotherapies to bolster host immune responses.

Innovations in GSH-centered therapies, CNS-targeted drug formulations, and vaccines that enhance GSH production in macrophages may significantly improve patient outcomes. However, further research is needed to validate these approaches. By integrating antimicrobial and immune-based strategies, including novel vaccines, future treatments may provide a comprehensive solution to combat MDR-TBM and improve the quality of life for affected patients.

## Figures and Tables

**Table 1:** Experimental Treatment Options for MDR-TBM.

Treatment Approaches	Components	Indications	Population Suitability	Key Considerations
Pretomanid-Based Combinations [23]	Pretomanid, Sutezolid, Moxifloxacin	Potential MDR-TBM treatment option, especially for cases needing CNS penetration	Experimental; currently tested in animal models with potential for human use	Higher brain tissue penetration than some MDR-TB drugs; promising animal model results
Modified WHO Regimens [23,24,25].	Bedaquiline, Pretomanid, Linezolid, Moxifloxacin	Adaptations for MDR-TBM under consideration, primarily developed for pulmonary MDR-TB	Patients with MDR-TB, experimental for CNS infections	CNS penetration is limited, requiring substitutions like sutezolid for effectiveness
Ongoing Research [22,26]	Various regimens in trials (e.g., endTB, TB-PRACTECAL)	Exploring short-course, all-oral regimens for MDR-TB with limited CNS adaptations	Focuses on general MDR-TB population; MDR-TBM suitability under investigation	Further research is essential for CNS efficacy, as current studies focus on pulmonary MDR-TB

MDR: Multidrug-Resistant, TB: Tuberculosis, CNS: Central Nervous System, WHO: World Health Organization.

**Table 2:** Challenges of Anti-TB Drugs in Treating MDR-TBM.

Drug	CNS Penetration	Safety and Challenges for MDR-TBM
Pretomanid [30,31]	Moderate CNS penetration; higher than some MDR-TB drugs	Limited data on safety in human TBM; potential for efficacy
Moxifloxacin [32,33,34]	Moderate CNS penetration; commonly used in TBM studies	Risk of QT prolongation; moderate CNS efficacy
Levofloxacin [32,33,34]	Moderate CNS penetration; some effectiveness in TBM cases	Risk of tendon damage; CNS efficacy moderate
Bedaquiline [28,29]	Low CNS penetration, reducing efficacy for TBM	Low efficacy in CNS; associated with hepatotoxicity
Delamanid [35]	Low CNS penetration; not optimal for TBM	Limited CNS efficacy; risk of QT prolongation
Linezolid [36,37,38,39]	Better CNS penetration but associated with neurotoxicity	Potential neurotoxicity; requires careful monitoring
Clofazimine [40]	Low CNS penetration; limited efficacy in the CNS	Associated with skin discoloration; low CNS effectiveness
Pyrazinamide [41]	Moderate CNS penetration; some effect in TBM	Myelosuppression risk; requires monitoring in TBM

CNS: Central Nervous System, MDR: Multidrug-Resistant, TB: Tuberculosis, QT: QT Interval.

**Table 3:** Mechanisms and Advantages of Immunotherapies in MDR-TBM.

Cytokine/Therapy	Mechanism of Action	Preclinical/Clinical Findings	How They Could Improve MDR-TBM
Recombinant IL-2 [44,45]	Enhances T-cell proliferation and activation; increases cytotoxic T lymphocyte response	Significant improvement in conversion rates in patients with MDR-TB receiving anti-TB therapy	Boosts adaptive immunity, enhancing clearance of Mtb and reducing treatment duration.
Recombinant IL-7 [46,47]	Promotes survival and proliferation of memory T cells; supports lymphocyte homeostasis	Statistically significant reduction in lung mycobacterial load in supplemented mice	Enhances long-term immunity and reduces risk of disease recurrence by maintaining T cell memory.
Recombinant IL-12 [48,49]	Activates macrophages and T cells; promotes Th1 polarization and IFN-γ production	Effectively reduced Mtb loads in mice; enhances immune response	Strengthens innate and adaptive immune responses, enhancing pathogen clearance and improving treatment outcomes.
Recombinant IL-15 [46,47]	Supports survival and activation of memory CD8+ T cells; enhances NK cell function	Enhanced survival in Mtb-infected mice	Increases the cytotoxic response against Mtb, aiding in the reduction in bacterial loads.
Recombinant IL-24 [50]	Activates CD8+ T cells to produce IFN-γ; enhances neutrophil function	Suppression of IL-24 may increase susceptibility and contribute to chronic TB	Promotes robust Th1 responses, potentially preventing chronic infection and improving outcomes.
IFN-γ (Intramuscular/Aerosolized) [51,52]	Enhances innate immune response; activates macrophages and increases antigen presentation	Significant improvement in chest radiograph outcomes in patients with pulmonary MDR-TB	Increases macrophage function and Mtb clearance, potentially shortening treatment time.
Imiquimod (TLR Agonist) [53]	Induces autophagy in macrophages; increases nitric oxide and pro-inflammatory cytokine production	Significantly inhibited Mtb growth in macrophages through autophagy	Enhances autophagic pathways to eliminate intracellular bacteria, improving immune responses against MDR-TB.
Anti-IL-4 Neutralizing Antibodies [54]	Shifts immune response toward Th1; reduces Th2-mediated suppression	Significantly reduced bacterial load in murine model, indicating improved immune response	Targeting Th2 responses may enhance Th1 activity, improving the effectiveness of TB treatment.
Corticosteroids [55]	Reduces inflammation and TNF-α levels; modulates immune response	Significant decrease in mortality risk and improved survival rates in TBM patients	Controls excessive inflammation, potentially improving the safety and efficacy of TB treatments.

MDR: Multidrug-Resistant, TB: Tuberculosis, IL: Interleukin, TLR: Toll-Like Receptor, TNF: Tumor Necrosis Factor, IFN: Interferon.

**Table 5:** Strategies to Enhance Glutathione for MDR-TBM Vaccines.

Strategy	Key Focus	Mechanism
Stimulating Nrf2 Activation [89,90,91]	Upregulate glutathione synthesis	Utilize adjuvants or immunostimulatory molecules (e.g., curcumin, sulforaphane) to activate Nrf2. This promotes the expression of genes involved in glutathione synthesis, such as glutamate-cysteine ligase and glutathione synthetase, increasing intracellular glutathione levels and enhancing ROS production.
Boosting Cysteine Levels [92]	Enhance intracellular cysteine availability	Incorporate elements that upregulate SLC7A11 (cystine/glutamate transporter) to improve cysteine import into macrophages. Increased cysteine availability facilitates the conversion to glutathione via the enzyme GCL, thus sustaining glutathione synthesis and enhancing oxidative stress against Mtb.
Sustaining Active Glutathione [93]	Maintain sufficient NADPH supply	Activate the pentose phosphate pathway to boost NADPH production by enhancing the expression of enzymes like glucose-6-phosphate dehydrogenase. This NADPH is crucial for maintaining glutathione in its reduced form, ensuring effective detoxification of ROS and enhancing macrophage oxidative responses against Mtb.

Nrf2: Nuclear Factor Erythroid 2–Related Factor 2, SLC7A11: Cystine/Glutamate Transporter, GCL: Glutamate-Cysteine Ligase, NADPH: Nicotinamide Adenine Dinucleotide Phosphate, ROS: Reactive Oxygen Species, Mtb: *Mycobacterium tuberculosis*.

## Data Availability

No new data were created or analyzed in this study. Data sharing is not applicable to this article.
